# Case Report: Recurrence of an Extradural Spinal Epidermoid Cyst Following Surgical Excision in a Dog

**DOI:** 10.3389/fvets.2022.871023

**Published:** 2022-04-15

**Authors:** Dillon Devathasan, Masahiro Murakami, Margaret A. Miller, Stephanie A. Thomovsky, Melissa J. Lewis

**Affiliations:** ^1^Department of Clinical Sciences, College of Veterinary Medicine, Auburn University, Auburn, AL, United States; ^2^Department of Veterinary Clinical Sciences, College of Veterinary Medicine, Purdue University, West Lafayette, IN, United States; ^3^Department of Comparative Pathobiology, College of Veterinary Medicine, Purdue University, West Lafayette, IN, United States

**Keywords:** congenital, keratin, extradural, epidermoid cyst, surgery

## Abstract

Congenital epidermoid cysts are slow-growing, mass lesions caused by the abnormal inclusion of neuroectodermal tissue within the developing central nervous system. Subtotal excision of epidermoid cysts increases the risk of early recurrence of clinical signs. A 4-year-old female spayed boxer was presented with a 4-month history of ambulatory paraparesis and proprioceptive ataxia. Neurological examination localized a T3-L3 myelopathy. MRI revealed a T1 iso- to hypointense, T2 and FLAIR hyperintense, rim-enhancing mass at the level of the T9-T10 vertebrae resulting in extradural compression of the spinal cord. This was histopathologically confirmed as an extradural epidermoid cyst following subtotal excision. MRI performed 2 months post-operatively revealed a significant decrease of the lesion volume. The dog was neurologically normal following the surgery however re-presented 28 months later with recurrence of clinical signs. A 28-month post-operative MRI revealed substantial enlargement of the epidermoid cyst. The dog was subsequently taken for repeat decompressive surgery. At 6 months from the repeat surgery, the dog was neurologically static with mild proprioceptive deficits. The case report highlights the clinical and MRI features of a recurrent extradural spinal epidermoid cyst treated by subtotal excision.

## Introduction

Epidermoid cysts are rare, mass lesions of the central nervous system (CNS) (1, 2). Congenital epidermoid cysts are most common and arise due to failure of separation of the neuroectoderm from the ectoderm during embryogenesis (2–4). This results in aberrant inclusion of epithelial tissue within the developing CNS (2). Less commonly, an acquired form can occur secondary to lumbar puncture, trauma or spinal surgery (5). In people, epidermoid cysts account for 1.8% of all intracranial and <1% of all intraspinal mass-lesions (1, 2). Intraspinal epidermoid cysts have been reported at all levels of the spinal cord in human medicine (6–10). Prevalence and location of intracranial and intraspinal epidermoid cysts have not been established in dogs with only sporadic case reports published (3, 4, 11–22). Four presumed congenital cases of intraspinal epidermoid cysts have been reported in medium to large breed dogs between the ages of one-and-a-half to 5 years (4, 17–19). The locations were thoracic (*n* = 3) or thoracolumbar (*n* = 1) and intramedullary in three and extradural in one (4, 17–19).

MRI is the modality of choice for diagnosis and surgical planning of epidermoid cysts (23, 24). Typical findings include a T2 and FLAIR hyperintense mass with peripheral rim contrast enhancement with varying levels of T1 hyperintensity due to intracapsular lipid content (3, 17, 22–24). MRI findings of a T9 intramedullary epidermoid cyst have been previously reported in a dog (17). There are, however, no reports documenting MRI changes following surgical excision. On gross examination, epidermoid cysts are well-defined, lobulated “pearly-white” masses consisting of a fibrous capsule containing fluid and keratinaceous debris (6, 25). Histopathologically, the cyst is lined by keratinizing stratified squamous epithelium and partially filled with keratin lamellae (desquamated epithelial cells from the stratum corneum), lipid, cholesterol, and occasional leukocytes (7, 19, 26).

In people, total surgical excision is the gold standard treatment for epidermoid cysts (7, 24). Subtotal excision is, however, more commonly achieved due to the intimate adherence of the capsule to the spinal cord (24). Unresected lesion and tumor debris increase the risk of early recurrence (7, 24). Among the three possible locations from which epidermoid cysts can arise (extradural, intradural-extramedullary or intramedullary), intramedullary lesions carry the worst prognosis due to poor intraoperative visualization and associated challenge of complete resection. Two veterinary case reports describe surgical excision of an intraspinal epidermoid cyst, intramedullary in one and extradural in the other (18, 19). For the intramedullary cyst, recurrence was confirmed at necropsy 4 months post-operatively while mild residual ataxia with no neurologic deterioration at 7 months post-operatively was reported for the extradural cyst (18, 19). To the author's knowledge, this is the first report describing the post-operative MRI and clinical findings in a dog with a recurrent extradural epidermoid cyst treated by repeat surgical excision.

## Case Presentation

A 4-year-old female spayed boxer was referred to the Purdue University Veterinary Hospital for a 4-month history of progressive paraparesis and pelvic limb ataxia. Neurologic examination revealed ambulatory paraparesis with severe proprioceptive ataxia and no associated pain on spinal palpation. Based on these findings, a T3-L3 myelopathy was localized. The patient was otherwise healthy on systemic screening (complete blood count, serum biochemistry, thoracic and abdominal radiographs).

**Figure 1:**
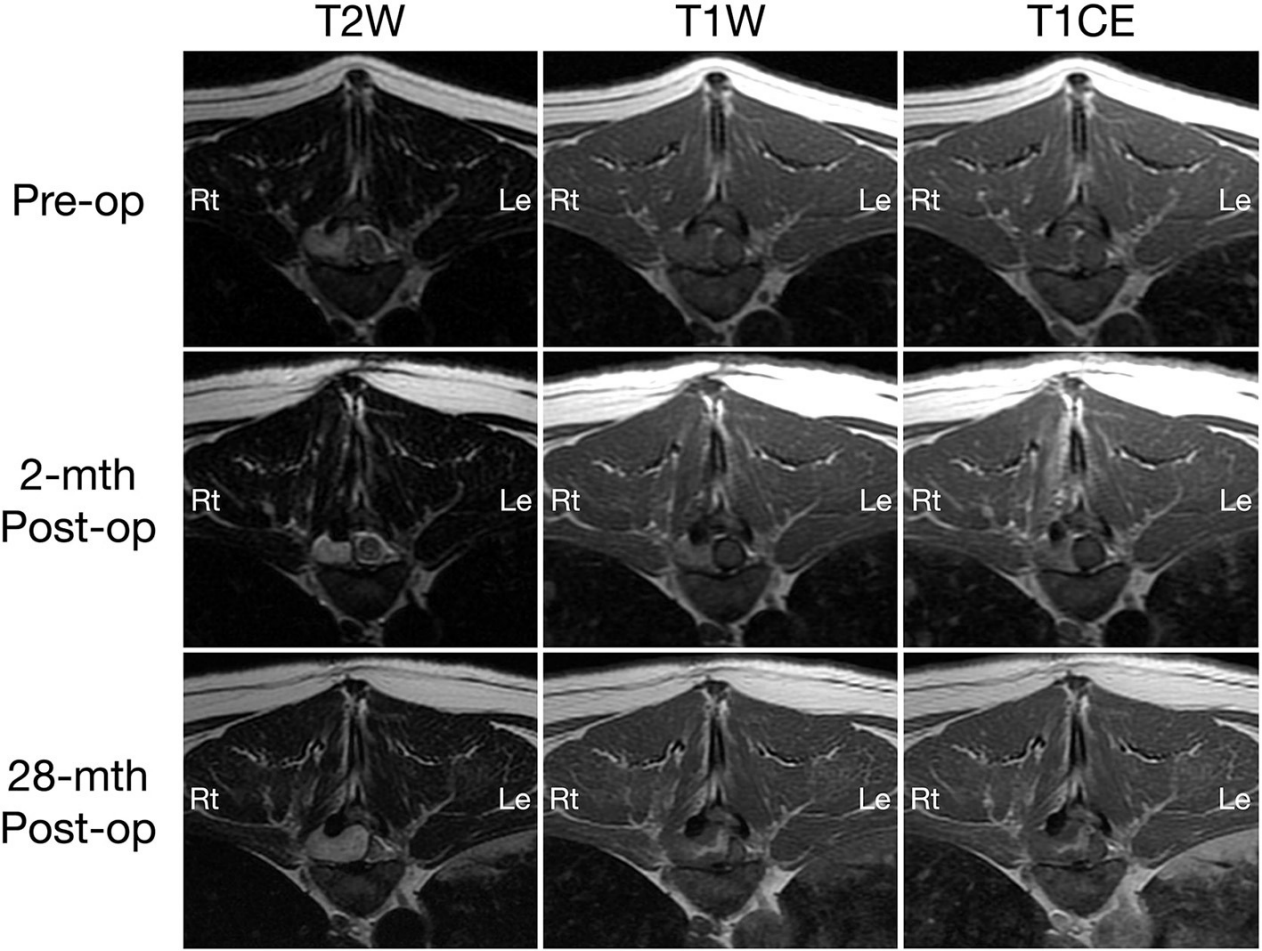
Transverse T2-weighted (left column) and pre- (middle column) and post-contrast T1W (right column) MRI images of a dog with extradural spinal epidermoid cyst; pre-operative (top row) and 2-month (middle row) or 28-month post-operative (bottom row) at the level of T9-T10 intervertebral disc space.

**Figure 2:**
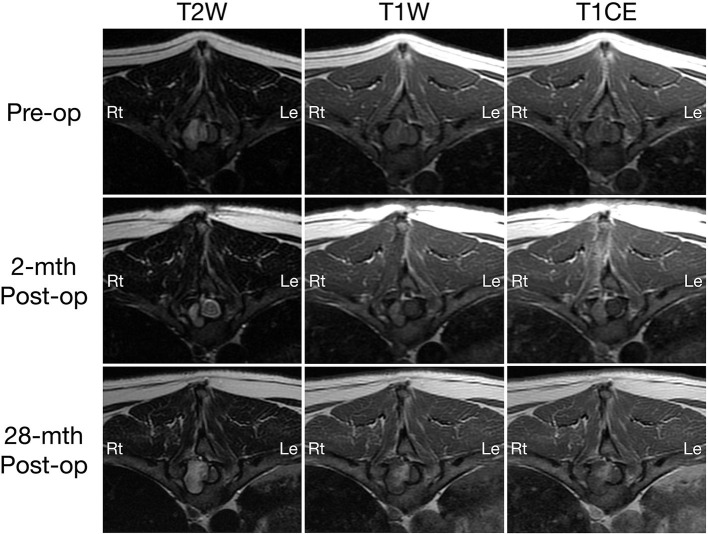
Transverse T2-weighted (left column) and pre- (middle column) and post-contrast T1W (right column) MRI images of a dog with extradural spinal epidermoid cyst; pre-operative (top row) and 2-month (middle row) or 28-month post-operative (bottom row) at the level of T10 vertebral body. The volume of the mass was reduced 2-month after surgery (middle column), but regrowth is observed 28 months post-surgery with progressive bone resorption and spinal cord compression (bottom row).

On MRI, a well-defined irregularly-shaped extradural mass with approximate height of 1.2 cm and craniocaudal length of 1.4 cm was present in the right lateral aspect of the spinal canal at the level of the T9-T10 intervertebral disc space, filling ~60% of the cross-sectional area with associated marked spinal cord compression (Figures 1, 2, top row). The mass extended cranially from the level of the T9 caudal vertebral endplate, caudally to the mid T10 vertebral body and into the right T9-T10 intervertebral foramen which was abnormal widened. The mass caused secondary resorption of the T10 vertebral body and right cranial articular process with smooth and rounded osseous margins. The mass was heterogeneously T2 and STIR hyperintense and T1 iso- to hypointense relative to the normal spinal cord parenchyma. On post-contrast T1-W images, there was a thin peripheral rim of mild enhancement. Differential diagnoses included neoplasia or a fungal granuloma (17).

Cerebrospinal fluid (CSF) was obtained from the cerebellomedullary cistern. Analysis revealed protein-cytological dissociation (protein −70 mg/dl (reference range 0–35 mg/dL), 655 red blood cells/μL and 2 nucleated cells/μL). Cytological evaluation demonstrated 6 large mononuclear cells and 3 non-degenerative neutrophils per high powered field with normal cytologic characteristics.

Following CSF collection, a T9-T10 right-sided hemilaminectomy and surgical debulking was performed. Dissection of the epaxial muscles revealed an irregular, firm, white fibrous mass adhered to the base of the T10 spinous process extending cranially to the T9-T10 articular facet. The mass extended into the spinal canal causing severe extradural spinal cord compression from caudal T9 to the mid aspect of T10. No overt capsule was visualized. Due to the firm adherence of lesion to the adjacent bone and poor visualization of the ventral extent of the mass into the T10 vertebral body, gross abnormal tissue was left *in situ*, though the spinal cord was adequately decompressed. Samples of the lesion were submitted for histopathology. No intra-operative complications were encountered.

**Figure 3:**
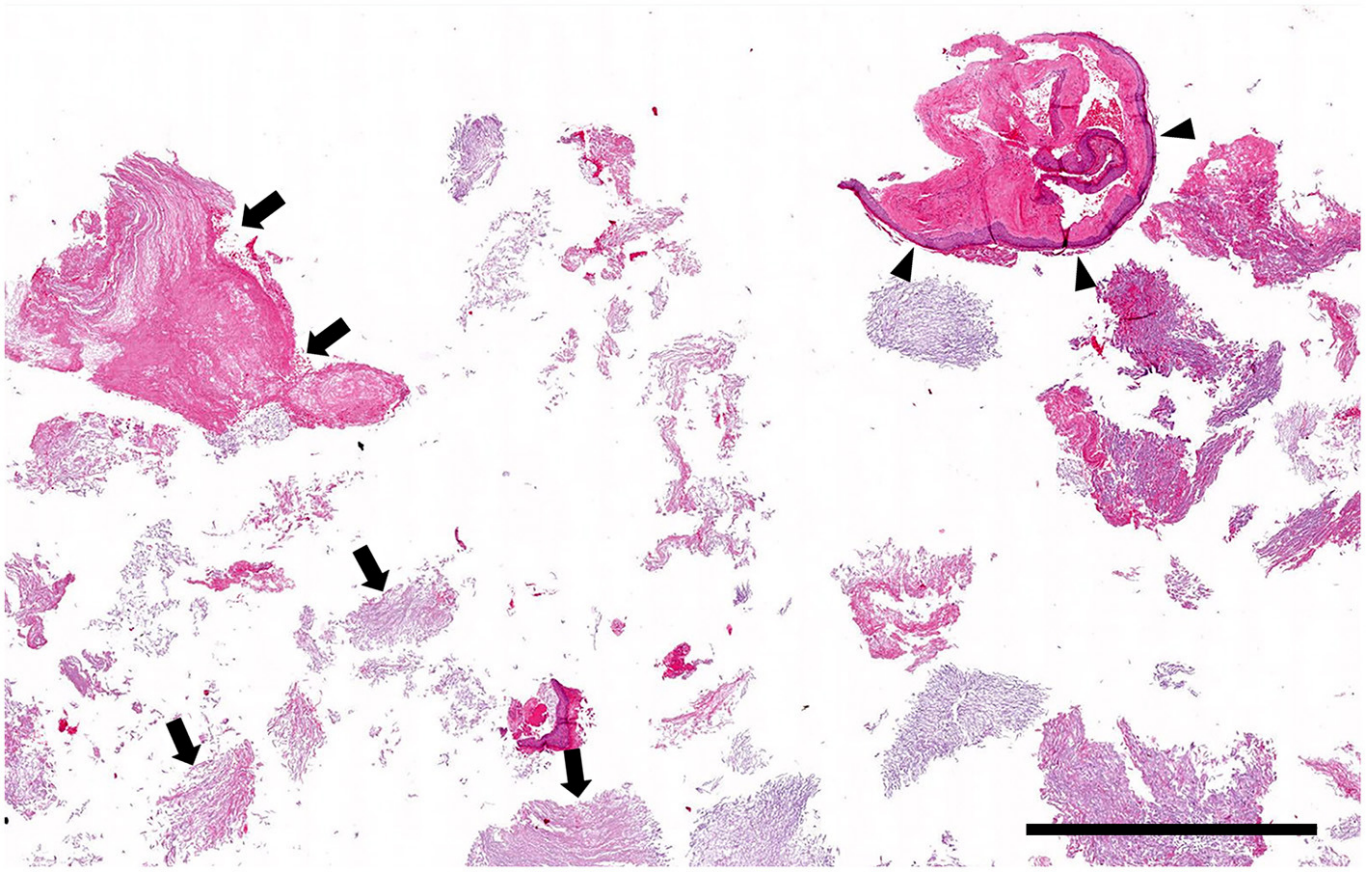
Histopathology low magnification (0.5 × objective): Cystic lesion. The biopsy specimen consists mainly of stacks of lamellar keratin (arrows) with only a few short segments of cyst lining (arrowheads). Hematoxylin and eosin stain; Bar = 1.5 mm.

**Figure 4:**
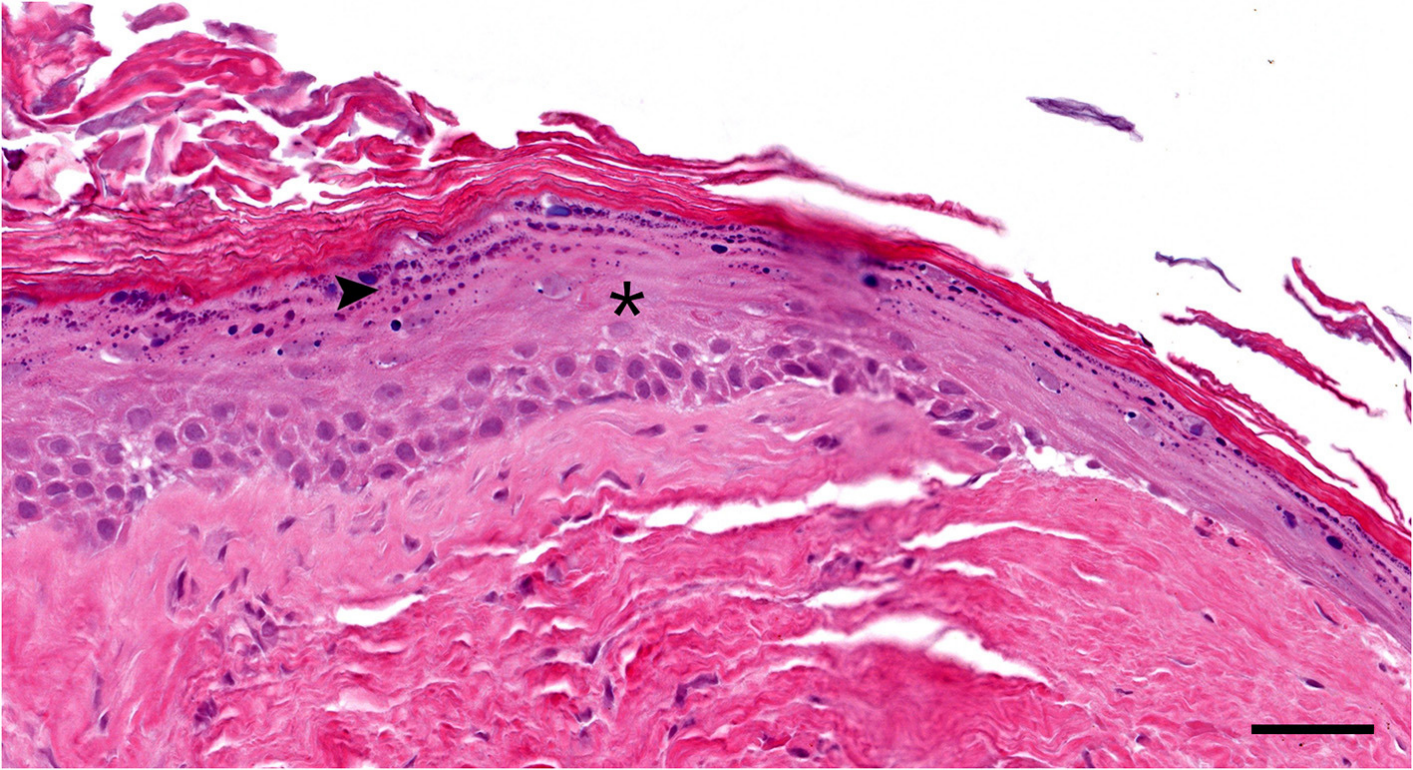
Histopathology high magnification (20 × objective): Segment of the cyst lining. The epidermis-like stratified squamous epithelium has thickened stratum spinosum (asterisk) and stratum granulosum (arrowhead) with orthokeratotic hyperkeratosis. Note the lack of hair follicles or adnexal glands in the underlying fibrous tissue Hematoxylin and eosin stain; Bar = 50 μm.

Histologically, the surgical biopsy specimens included stacks of orthokeratotic lamellar keratin and a few 2-mm-long segments of keratinizing stratified squamous epithelium supported by fibrous tissue without adnexa (Figures 3, 4). The epithelium was 6–12 cell layers thick with normal maturation from stratum basale through stratum spinosum, stratum granulosum and orthokeratotic stratum corneum. The finding of epidermis-like stratified squamous epithelium without cutaneous adnexa was the basis for the diagnosis of epidermoid cyst.

In addition to routine postoperative analgesia, the patient was placed on 1 mg/kg/day of prednisone. Daily neurological evaluations were performed over the 5-day period of hospitalization. The patient was ambulatory at time of discharge with improved paraparesis and moderate proprioceptive ataxia.

A 2-month post-operative MRI revealed a decrease in size of the epidermoid cyst (approximate height of 1.0 cm and craniocaudal length of 1.3 cm) in the spinal canal at the level of the T9-T10 intervertebral disc space with minimal spinal cord compression (Figures 1, 2, middle row). The mass remained present in the right lateral aspect of the spinal canal and extended through the T9-T10 intervertebral foramen and into the right dorsal aspect of the T10 vertebral body defect. The mass was T2 and STIR hyperintense however was now noted to be mildly T1 heterogeneously hyperintense. A focal, ill-defined T2 hyperintense, non-enhancing intramedullary lesion was observed at the level of T10. Additional bony changes were consistent with static perilesional pressure resorption and the prior T9-T10 hemilaminectomy.

By 12-weeks post-operatively, the patient was minimally ataxic. The patient re-presented 28 months later for a 3-week history of progressive paraparesis and ataxia. On neurological examination, the patient was strongly ambulatory with mild proprioceptive ataxia. Proprioceptive paw placement was delayed in the left pelvic limb. The remainder of the neurological examination was unremarkable.

Compared to the 2-month post-operative scan, repeat 28-month post-operative MRI demonstrated substantial enlargement of the epidermoid cyst (approximate height of 1.5 cm and craniocaudal length of 1.6 cm) which occupied ~70–80% of the right lateral to right dorsolateral aspect of the T9-T10 spinal canal. This resulted in severe extradural spinal cord compression with progressive extension and bony resorption of the right dorsal aspect of the cranial T10 vertebral body (Figures 1, 2, bottom row). The lesion was noted to be irregularly-shaped on T2 W images and ill-defined with T1 heterogeneous hyperintensity and no contrast enhancement. The rest of the MRI features remained unchanged from the previous scan including a persistent intramedullary T2 hyperintensity adjacent to the mass.

At 28-months following the initial surgery, a repeat decompressive surgery was performed. There was extensive, highly vascular fibrous scar tissue overlying the T8-T11 vertebrae. The previous T9-T10 hemilaminectomy site was challenging to visualize. A right hemilaminectomy was performed at T11-12 to visualize normal spinal cord with subsequent cranial extension to the level of T8-T9. Fibrous adhesions at the previous T9-T10 hemilaminectomy window were sharply dissected with an #11 blade to expose spinal cord. The primary mass was a pale gray opalescent structure. Surrounding the mass at the cranial and caudal margins and extending into the T10 vertebral body was dark brown to black granular material, suspected to be keratin or necrotic epithelial material. The mass and granular material were debulked including the extension into the dorsal aspect of T10 vertebral body. No grossly visible abnormal tissue remained, and the spinal cord was adequately decompressed prior to routine closure of the surgical site. Samples of the lesion were submitted for histopathology with similar findings as for the original biopsy specimen and a diagnosis of epidermoid cyst. No intra-operative complications were encountered. The patient was discharged 4 days post-operatively and was strongly ambulatory with mild proprioceptive ataxia. The owner was contacted 6 months after the second surgery and reported that the patient was neurologically static; the dog remained strongly ambulatory with mild proprioceptive ataxia.

## Discussion

This case report highlights the clinical and imaging features of repeat subtotal resection in a recurrent intraspinal extradural epidermoid cyst in a dog. Surgical excision is considered the gold standard of treatment since epidermoid cysts are neither responsive to radiation nor chemotherapy (24, 27). The goal of surgical intervention is total excision of the lesion while preserving neurological function (28). Ease of achieving total excision corresponds to the relative location of the epidermoid cyst to the spinal cord (i.e. extradural, intradural-extramedullary or intramedullary) (7, 28). Extradural and intradural-extramedullary cysts are easier to visualize compared to intramedullary lesions as a clear plane of dura or arachnoid membrane, respectively, separates the lesion and spinal cord (7). More commonly, subtotal excision is achieved due to tight adherence of the capsule to the spinal cord and/or surrounding structures (7, 24). Subtotal excision substantially increases the risk of tumor regrowth (1, 7, 24).

In the dog reported here, complete resection was not performed in either surgery. However, the extradural location facilitated sufficient decompression of the spinal cord and associated clinical improvement. Following both surgeries, the patient showed a significant improvement immediately post-operatively. Relapse of clinical signs secondary to substantial cyst regrowth occurred 28 months after the first surgery with no deterioration of clinical status noted at 6 months after the second subtotal resection. A single case report describes surgical excision of an extradural epidermoid cyst in a boxer which was minimally affected 7 months post-operatively, but longer-term follow-up was not reported (19). There are no reports of repeat surgical excision in dogs. In three case reports of intramedullary epidermoid cysts in dogs, two were euthanized at diagnosis and one 4 months after surgery (4, 17, 18). However, resection of intramedullary epidermoid cysts in people carries a poor prognosis due to the delicate nature of the surgery and increased risk of permanent damage to neural tissue (7, 24). The findings in our dog support the potential for long-term improvement in neurologic function for an extradurally-located epidermoid cyst, especially if re-operation is performed at recurrence.

A reported risk of subtotal excision is aseptic meningitis secondary to rupture of cystic contents. Free fatty acids and cholesterol accumulate within the cavity due to inflammation associated with necrosis of the degenerating squamous cells (5, 22). Following cyst rupture, cystic contents that enter the subarachnoid space or contact the meninges can produce a granulomatous or chemical meningitis attributed to fatty acids and cholesterol (6). In people, cystic rupture has been reported with extradural, intradural-extramedullary and intramedullary locations and has been associated with gradual onset of weakness or occasionally acute paraplegia (29, 30). Therefore, drainage of cystic contents prior to surgical debulking is a routine strategy in people to minimize the risk of cystic rupture (24). In our canine patient, an obvious cystic capsule was not visualized in either surgery hence cystic drainage was not performed. Disruption of the cystic lining might have occurred prior to presentation or inadvertently during debulking. Regardless, there were no clinical signs associated of aseptic meningitis in this dog. A tapering course of steroids after both surgeries might have reduced inflammation and minimized the risk of aseptic meningitis. It is also possible cyst rupture in an extradural location is less likely to result in meningitis compared to intradural lesions.

Although congenital in origin, due to their slow-growing nature, epidermoid cysts are often asymptomatic in their early stages (24). In people, clinical presentation occurs between the 2nd to 5th decades of life with a mean presentation age of 34 years (25). Average time between onset of symptoms and diagnosis is 6 years (25). A study by Alvord demonstrated that the growth of an epidermoid cyst is linear as opposed to exponential which is seen in most neoplasms (8). In our patient, clinical signs began at 4 years and 3 months of age with progression over a 4-month period. A congenital lesion was suspected in our patient as there was no history of trauma or spinal tap to implicate an iatrogenic etiology. Onset of neurologic deficits in other dogs has been reported between one-and-a-half to 5 years of age with progression ranging from acute to slowly progressive (up to 6 months) (4, 17–19). A plausible reason for the later onset of clinical signs and long interval between recurrences in this dog is the extradural location of the epidermoid cyst. As compared to an intramedullary-located lesion, an extradural location may accommodate for greater capacity of outward expansion before impinging on spinal cord.

On MRI, epidermoid cysts show variable signal intensity based on different components of the tumor (2). Typical imaging characteristics include T2 and FLAIR hyperintensity similar to that of CSF (due to intracapsular cystic fluid), variable T1 iso-hypointensity based on intracapsular lipid content and contrast enhancement of the epithelial lining on post-contrast T1-W images (3, 11, 17, 22, 23, 31). On the initial scan, the lesion in our patient shared the aforementioned MRI characteristics. Notably, the lesion in our dog was T1 hyperintense following the first surgery. In people, T1-hyperintensity associated with epidermoid cysts is an atypical finding but has been attributed to high cystic protein content (>9.0g per 100mL), calcification of keratinized debris or intracystic hemorrhage (paramagnetic effect) (28).

Another notable change on the follow-up scans was the presence of a focal intramedullary perilesional T2-hyperintensity. In intramedullary epidermoid cysts, perilesional spinal cord changes have been attributed to leakage of cystic content resulting in inflammation and gliosis along the margin of the lesion (32). Given the extradural location of the lesion in this case, cystic rupture is considered an unlikely explanation. Perilesional edema is not a typical feature of spinal epidermoid cysts in people but has been reported in veterinary literature (2, 17, 32). A T2-hyperintensity cranial to an epidermoid cyst in a 5 year-old female spayed mastiff was histopathologically consistent with edema at autopsy (17). A plausible explanation for the intramedullary lesion noted on follow-up imaging in this dog is gliosis or edema secondary to spinal cord manipulation during surgery or residual compression. Gliosis is a well-documented intramedullary change following surgery or trauma in cases such as intervertebral disc herniation (33).

In addition to the changes within the spinal cord noted on imaging, our patient demonstrated osseous changes in the region of the mass with overlapping characteristics to an extradural epidermoid cyst previously reported in another boxer (19). Both patients had lesions extending from the external vertebral surface into the intracanal space, dural ossification and sclerosis of perilesional bone. The dog reported here also showed widening of intervertebral foramen and pressure resorption of the adjacent vertebra. These changes likely reflect the extradural location of these masses since osseous changes have not been described in veterinary cases of intramedullary epidermoid cysts (4, 17, 18). In people, osseous change appears to be infrequently reported in intraspinal epidermoid cysts with only two documented cases at the intradural-extramedullary level (34, 35). Although an underlying pathophysiology has not been established, MRI characteristics suggest that chronic expansion of the epidermoid cyst results in pressure resorption and remodeling of adjacent bone.

## Conclusion

In summary, we report the surgical removal of a recurrent intraspinal extradural epidermoid cyst in a dog. MRI is the modality of choice for diagnosis and surgical planning of the lesion. Although signal intensity on T1W images can vary, an epidermoid cyst should be considered as a differential diagnosis for a T2 and FLAIR hyperintense, peripherally contrast-enhancing mass lesion is observed on MRI in a young to middle-aged canine. Despite subtotal excision, there was a long interval before relapse of clinical signs and improvement with re-operation. This case supports that dogs with extradural epidermoid cysts have a better outcome than previously reported dogs with intramedullary epidermoid cysts. It is therefore likely that the prognosis for spinal epidermoid cysts is dictated by the extent of surgical removal and location relative to the spinal cord. Finally, recurrence of clinical signs can be expected following subtotal resection and regular follow-up MRI scans and examinations are recommended.

## Data Availability Statement

The original contributions presented in the study are included in the article, further inquiries can be directed to the corresponding author/s.

## Ethics Statement

Ethical review and approval was not required for the animal study because written informed consent was obtained from the owners for the participation of their animals in this study.

## Author Contributions

DD contributed to the writing of the manuscript and literature review. MAM contributed to the histopathological examination, interpretation, and description of biopsy samples. MM contributed to the interpretation and preparation of MRI images. DD, ML, and ST contributed to the preparation of the manuscript. All authors reviewed and edited the manuscript.

## Conflict of Interest

The authors declare that the research was conducted in the absence of any commercial or financial relationships that could be construed as a potential conflict of interest.

## Publisher's Note

All claims expressed in this article are solely those of the authors and do not necessarily represent those of their affiliated organizations, or those of the publisher, the editors and the reviewers. Any product that may be evaluated in this article, or claim that may be made by its manufacturer, is not guaranteed or endorsed by the publisher.

## References

[1] Barbagallo GMV Maione M Raudino G Certo F. Thoracic intradural-extramedullary epidermoid tumor: the relevance for resection of classic subarachnoid space microsurgical anatomy in modern spinal surgery. Technical Note and Review of the Literature. World Neurosurg. (2017) 108:54–61. 10.1016/j.wneu.2017.08.078 28843754

[2] Amato V Assietti R Arienta C. Intramedullary epidermoid cyst: Preoperative diagnosis and surgical management after MRI introduction. J Neurosurg. (2002) 46:122–6. 12690335

[3] Mackillop E. Magnetic resonance imaging of intracranial malformations in dogs and cats: intracranial malformations. Vet Radiol Ultrasound. (2011) 52:S42–51. 10.1111/j.1740-8261.2010.01784.x 21392155

[4] Tomlinson J Higgins R LeCouteur R Knapp D. Intraspinal epidermoid cyst in a dog. J Am Vet Med Assoc. (1988) 193:1435–6. 3209460

[5] Gardner DJ O'Gorman AM Blundell JE. Intraspinal epidermoid tumour: late complication of lumbar puncture. CMAJ. (1989) 141:223–5. 2752348 PMC1269411

[6] Gonzalvo A Hall N McMahon JHA Fabinyi GC. Intramedullary spinal epidermoid cyst of the upper thoracic region. J Clin Neurosci. (2009) 16:142–4. 10.1016/j.jocn.2008.04.017 19013801

[7] Liu H Zhang J-N Zhu T. Microsurgical treatment of spinal epidermoid and dermoid cysts in the lumbosacral region. J Clin Neurosci. (2012) 19:712–7. 10.1016/j.jocn.2011.07.046 22436108

[8] Scarrow AM Levy EI Gerszten PC Kulich SM Chu CT Welch WC. Epidermoid cyst of the thoracic spine: case history. Clin Neurol Neurosurg. (2001) 103:220–2. 10.1016/S0303-8467(01)00156-1 11714565

[9] Nica DA Strambu VE Roşca T Copaciu R Stroi M Ciurea AV. Popa F. Acquired epidermoid cysts of the cauda equine. J Med Life. (2011) 4:305–9. 22567058 PMC3168827

[10] Sharifi G Mousavinejad SA Dehghan M Khosravi YD Ebrahimzadeh K Samadian M. Rare Intramedullary epidermoid cyst of the thoracic spinal cord: case report and review of literature. Iran J Neurosurg. (2018) 4:225–32. Available online at: http://irjns.org/article-1-155-en.html&sw=Intramedullary+Epidermoid+Cyst

[11] Steinberg T Matiasek K Brühschwein A. Fischer A. Imaging diagnosis—intracranial epidermoid cyst in a Doberman Pinscher. Vet Radiol Ultrasound. (2007) 48:250–3. 10.1111/j.1740-8261.2007.00238.x 17508513

[12] De Decker S Davies E Benigni L Wilson H Pelligand L Rayner EL. Surgical treatment of an intracranial epidermoid cyst in a dog. Vet Surg. (2012) 41:766–71. 10.1111/j.1532-950X.2012.01010.x 22759067

[13] Zaki FA. Spontaneous central nervous system tumors in the dog. Vet Clin North Am. (1977) 7:153–63. 10.1016/S0091-0279(77)50013-5 191973

[14] Kawaminami A Tawaratani T Nakazawa M Uchimoto H Sumi N. A case of multiloculated, intracranial epidermoid cyst in a beagle dog. Lab Anim. (1991) 25:226–7. 10.1258/002367791780808284 1921319

[15] Kornegay JN. Gorgacz EJ. Intracranial epidermoid cysts in three dogs. Vet Pathol. (1982) 19:646–50. 10.1177/030098588201900608 6983175

[16] Platt SR Graham J Chrisman CL Adjiri-Awere A. Clemmons RM. Canine intracranial epidermoid. cyst Vet Radiol Ultrasound. (1999) 40:454–8. 10.1111/j.1740-8261.1999.tb00374.x 10528837

[17] Lipitz L Rylander H. Pinkerton ME. Intramedullary epidermoid cyst in the thoracic spine of a dog. J Am Anim Hosp Assoc. (2011) 47:e145–9. 10.5326/JAAHA-MS-5583 22058362

[18] Shamir MH Lichovsky D Aizenberg I Chrisman CL. Partial surgical removal of an intramedullary epidermoid cyst from the spinal cord of a dog. J Small Anim Pract. (1999) 40:439–42. 10.1111/j.1748-5827.1999.tb03119.x 10516951

[19] Cappello R Lamb CR Rest JR. Vertebral epidermoid cyst causing hemiparesis in a dog. Vet Rec. (2006) 158:865–7. 10.1136/vr.158.25.865 16798956

[20] O'Brien D Jergens A Nelson S. Intracranial epidermoid (cholesteatoma) associated with aseptic suppurative meningoencephalitis in an aged dog. J Am Anim Hosp Assoc. (1990) 26:582–5.

[21] Cherubini GB Mantis P Martinez TA Lamb CR Cappello R. Utility of magnetic resonance imaging for distinguishing neoplastic from non-neoplastic brain lesions in dogs and cats. Vet Radiol Ultrasound. (2005) 46:384–7. 10.1111/j.1740-8261.2005.00069.x 16250394

[22] MacKillop E Schatzberg SJ De Lahunta A. Intracranial epidermoid cyst and syringohydromyelia in a dog. Vet Radiol Ultrasound. (2006) 47:339–44. 10.1111/j.1740-8261.2006.00150.x 16863050

[23] Bloomer CW Ackerman A Bhatia RG. Imaging for spine tumors and new applications. Top Magn Reson Imaging. (2006) 17:69–87. 10.1097/RMR.0b013e31802bb38f 17198224

[24] Yin H Zhang D Wu Z Zhou W Xiao J. Surgery and outcomes of six patients with intradural epidermoid cysts in the lumbar spine. World J Surg Oncol. (2014) 12:50. 10.1186/1477-7819-12-50 24589060 PMC3975861

[25] Roux A Mercier C Larbrisseau A Dube LJ Dupuis C Del Carpio R. Intramedullary epidermoid cysts of the spinal cord. Case report J Neurosurg. (1992) 76:528–33. 10.3171/jns.1992.76.3.0528 1738035

[26] Lunardi P Missori P Gagliardi FM Fortuna A. Epidermoid tumors of the 4th ventricle: report of seven cases. Neurosurgery. (1990) 27:532–4. 10.1097/00006123-199010000-00004 2234353

[27] Cobbs CS Pitts LH Wilson CB. Epidermoid and dermoid cysts of the posterior fossa. Clin Neurosurg. (1997) 44:511–28. 10080024

[28] Lim J Cho K. Epidermoid cyst with unusual magnetic resonance characteristics and spinal extension. World J Surg Oncol. (2015) 13:240. 10.1186/s12957-015-0651-1 26245481 PMC4527251

[29] Munshi A Talapatra K Ramadwar M Jalali R. Spinal epidermoid cyst with sudden onset of paraplegia. J Can Res Ther. (2009) 5:290–2. 10.4103/0973-1482.59913 20160364

[30] Kim KY Kang JH Choi DW Lee MH Jang JH. Paraplegia due to Spinal Epidermoid Cyst Rupture at Asthma Attack. Ann Rehabil Med. (2013) 37:274–9. 10.5535/arm.2013.37.2.274 23705125 PMC3660491

[31] Dutt SN Mirza S Chavda SV Irving RM. Radiologic differentiation of intracranial epidermoids from arachnoid cysts. Otol Neurotol. (2002) 23:84–92. 10.1097/00129492-200201000-00019 11773853

[32] Chandra PS Manjari T Devi BI Chandramouli BA Srikanth SG Shankar SK. Intramedullary spinal epidermoid cyst. Neurol India. (2000) 48:75–7. 10751819

[33] Alisauskaite N Spitzbarth I Baumgärtner W Dziallas P Kramer S Dening R. Tipold A. Chronic post-traumatic intramedullary lesions in dogs, a translational model. PLoS ONE. (2017) 12:e0187746. 10.1371/journal.pone.0187746 29166400 PMC5699804

[34] Li J Qian M Huang X Zhao L Yang X Xiao J. Repeated recurrent epidermoid cyst with atypical hyperplasia: A case report and literature review. Medicine (Baltimore). (2017) 96:e8950. 10.1097/MD.0000000000008950 29245264 PMC5728879

[35] Ziv ET Gordon McComb J Krieger MD Skaggs DL. Iatrogenic intraspinal epidermoid tumor: two cases and a review of the literature. Spine (Phila Pa 1976). (2004) 29:E15–8. 10.1097/01.BRS.0000104118.07839.44 14699293

